# Combination of lapatinib with isothiocyanates overcomes drug resistance and inhibits migration of HER2 positive breast cancer cells

**DOI:** 10.1007/s12282-016-0700-9

**Published:** 2016-05-06

**Authors:** Angelika Kaczyńska, Anna Herman-Antosiewicz

**Affiliations:** Department of Molecular Biology, Faculty of Biology, University of Gdańsk, Wita Stwosza 59, 80-308 Gdańsk, Poland

**Keywords:** HER2, Isothiocyanates, Erucin, Lapatinib resistance

## Abstract

**Background:**

Lapatinib is a commonly used drug that interrupts signaling from the epidermal growth factor receptors, EGFR and HER2/neu. Long-term exposure to lapatinib during therapy eliminates cells that are sensitive to the drug; however, at the same time it increases probability of lapatinib-resistant cell selection. The aim of this study was to verify whether combinations of lapatinib with one of isothiocyanates (sulforaphane, erucin or sulforaphene), targeting different levels of HER2 signaling pathway, exert stronger cytotoxic effect than therapy targeting the receptor only, using heterogeneous populations consisting of lapatinib-sensitive and lapatinib-resistant breast cancer cells.

**Methods:**

Lapatinib-sensitive HER2 overproducing SKBR-3 breast cancer cells and their lapatinib-resistant derivatives were combined at different proportions to simulate enrichment of cancer cell population in a drug-resistant fraction during lapatinib therapy. Effects of treatments on cell survival (MTT), apoptosis induction (PARP cleavage), prosurvival signaling (p-Akt, p-S6) as well as cell motility (wound healing assay) and invasion (Boyden chamber assay) were investigated.

**Results:**

Combination of lapatinib with any of isothiocyanates significantly decreased cell viability and inhibited migration of populations consisting of different amounts of drug-sensitive and drug-resistant cells. In case of population entirely composed of lapatinib-resistant cells the most effective was combination of lapatinib with erucin which decreased cell viability and motility, phosphorylation of Akt, S6 and VEGF level more efficiently than each agent alone.

**Conclusions:**

Combination of lapatinib and isothiocyanates, especially erucin, might be considered as an effective treatment reducing metastatic potential of breast cancer cells, even these with the drug resistance phenotype.

## Introduction

Overproduction of HER2 (human epidermal growth factor receptor 2) is reported in nearly 20–25 % of all breast cancer cases. Enhanced signal transduction from amplified receptors, especially through Akt-mTOR-S6K signaling pathway, leads to uncontrolled proliferation, evasion of apoptosis, neoangiogenesis and enhanced cell motility, which may lead to metastasis. It has been reported that 30–40 % of women with breast cancer develop a metastatic disease [1], and that it may be associated with *HER2* gene amplification [2]. Since overproduction of HER2 correlates with poor clinical outcomes, this receptor became a target for anticancer therapies.

Currently, several treatments targeting HER2 are approved, which include monoclonal antibodies and small molecule inhibitors of receptor kinases or their combinations with chemotherapeutic agents [3, 4]. Trastuzumab (Herceptin; Genentech, South San Francisco, CA, USA) was the first monoclonal antibody developed to target HER2. It significantly improved outcomes for patients diagnosed with this subtype of cancer [5]. However, de novo or acquired resistance to trastuzumab eventually occurs in most patients with advanced disease [6]. Thus, trastuzumab is typically combined with chemotherapy to increase efficacy, which also increases toxicity. Additional HER2 targeting agents have been developed recently, such as pertuzumab or trastuzumab emtansine. Pertuzumab (Perjeta, Genentech) is a fully humanized monoclonal antibody that binds to a different epitope of the HER2 extracellular domain than trastuzumab, and prevents dimerization of the HER receptors [7]. It is effective in combination with trastuzumab and docetaxel in advanced breast cancers [8]. In 2013, the FDA approved the first successful HER2-targeted antibody–drug conjugate, trastuzumab emtansine (T-DM1; Kadcyla; Genentech), for the treatment of HER2-positive trastuzumab-pretreated advanced breast cancer. This drug inhibits HER2 signaling and is cytotoxic to HER2-positive cells due to emtansine which disrupts dynamics of microtubules [9]. T-DM1 appears to have some activity against central nervous system metastases [10].

Another class of agents used to block Her-2 signaling are small molecule tyrosine kinase inhibitors, such as lapatinib (Tykerb; GlaxoSmithKline, Brentford, United Kingdom). Mechanism of its action relies on blocking of the ATP-binding site in the cytoplasmic domain of HER2 which leads to inhibition of signal transduction cascade from the receptor [4]. In spite of a great success in breast cancer therapy, primary or acquired resistance to lapatinib still occurs, even when this medicament is used in combination with other commercially available anti-HER2 agents (e.g., trastuzumab) [11]. Long-term exposure to lapatinib causes elimination of drug-sensitive cells and at the same time increases probability of selection of lapatinib-resistant cells whose percentage increases in the cell population with time. Cancer cells respond to the first stage of treatment and tumor decreases, however, appearance of resistant cells, which typically occurs within 12 months of the start of therapy, may lead to progression of the disease and to metastasis [12, 13]. Many different mechanisms underlying this phenomenon have been proposed, including hyperactivation of the signaling network downstream of HER2 [14].

Overexpression of HER2 is also associated with vascular endothelial growth factor (VEGF) upregulation [15]. It has been shown that most types of human cancer cells overexpress VEGF and its receptor [16]. VEGF is one of the most important factors stimulating angiogenesis, which is essential for the growth of solid tumors. VEGF also induces expression of matrix metalloproteinases (MMPs) that degrade the basement membrane, thus it is also involved in first stages of endothelial cell migration and metastasis of cancer cells [17].

Recently, we demonstrated that isothiocyantes, such as sulforaphane (SFN), erucin (ERN) and sulforaphene (SF), enhance anti proliferative activity of lapatinib in HER2-positive breast cancer cells which was connected with a more efficient inhibition of pro survival signaling and induction of apoptosis [18]. Isothiocyanates (ITC) are naturally occurring compounds in *Brassicaceae* vegetables. They possess chemopreventive activities—inhibit phase I enzymes, which are responsible for carcinogens activation, and induce phase II enzymes that are involved in carcinogen elimination [19, 20]. Moreover, isothiocyanates reveal anticancer activity causing apoptosis through induction of oxidative stress and modulation of numerous cell signaling cascades which are crucial for cancer cell survival [21]. It has been previously shown that SFN inhibits Akt-mTOR survival pathway in leukemia cells [22] and in breast cancer cells differing in growth factor receptor status [23]. Importantly, there are reports showing that SFN more efficiently inhibits growth of human breast cancer cells than normal breast epithelial cells [24, 25].

Numerous reports indicate that isothiocyanates influence different signal transduction pathways downstream of growth factor receptors. For instance, Wu et al. suggested that phenethyl isothiocyanate (PEITC) and benzyl isothiocyanate (BITC) inhibited cell survival signaling kinase Akt, and suppressed lung cancer cell metastasis potential [26]. SFN, one of the best characterized ITC present in high concentrations in broccoli, decreased phosphorylation of Akt and S6K1 in MDA-MB-231, MCF-7, MDA-MB- 468 and SKBR-3 breast cancer cell lines [23] and inhibited Akt and mTOR pathway in acute lymphoblastic leukemia cells [22]. Moreover, in PC-3 prostate cancer cells, SFN inhibited activation of NF-κB, Akt and ERK which are all involved in cancer survival and metastasis [27]. Furthermore, in vivo, SFN inhibited the activation of MMPs and lung metastasis induced by melanoma cells in mice [28]. It has been also shown that SFN suppressed capillary-like tube formation on basement membrane matrix and inhibited HMEC-1 cell migration by down regulation of the vascular endothelial growth factor (VEGF) and its receptor KDR/flk-1, hypoxia-inducible factor-1α, c-Myc and matrix metalloproteinase 2 [29].

In this work we asked a question whether combination of lapatinib with sulforaphane, sulphoraphene or erucin overcomes development of the drug-resistant populations and whether such therapy protects against development of metastatic breast cancer. We have chosen lapatinib, because—in contrast to trastuzumab and its derivatives—it is orally bioavailable drug. Additionally, as a small molecule it is better suited to cross the blood–brain barrier, which rationalizes its use in patients with metastases to central nervous system for which HER2-positive tumors have predilection [30].

To answer these questions, we designed an in vitro simulation of the drug-dependent selection of resistant cells during lapatinib treatment and evaluated anticancer efficiency of combined therapy as compared to lapatinib or each of ITCs as the only agents. Lapatinib-sensitive and lapatinib-resistant SKBR-3 cells were combined in different proportions to simulate the heterogeneity of cancer cell populations and were treated with low doses of isothiocyanates (close to their IC_50_), the drug (below its IC_50_ for the sensitive cell line) or combinations of compounds. Their survival and migration potential was evaluated. As erucin-lapatinib combination was the most effective, we verified its influence on cell motility, invasion, VEGF level and HER2 downstream signaling.

## Materials and methods

### Reagents and cell lines

Lapatinib ditosylate (purity > 99.5 %), erucin (purity ≥ 99 %), S-sulforaphene (purity ≥ 99.7 %) and R,S-sulforaphane (purity ≥ 99 %) were obtained from LKT Laboratories (St. Paul, MN, USA). DMSO, thiazolyl blue tetrazolium bromide (MTT), the anti-β-actin, anti-mouse and anti-rabbit antibodies conjugated with HRP were from Sigma (St. Louis, MO, USA). The antibodies against p-Akt (Ser-473), Akt, p-S6 (Ser 235), PARP-1, VEGF from Santa Cruz Biotechnology (Santa Cruz, CA, USA) and p-HER2 (Tyr121/1222), HER2, S6 were from Cell Signaling Technology (Danvers, MA, USA).

Lapatinib-resistant SKBR-3 cells (LapR) were developed by a long-term exposure of SKBR-3 cells to increasing concentrations of the drug. Cells were cultured in RPMI 1640 medium supplemented with 10 % fetal bovine serum and 1 % penicillin/streptomycin mixture (Life Technologies, Carlsbad, CA, USA). The invasive potential of the cells was determined using BD BioCoat™ BD MatrigelTM Invasion Chamber (BD Biosciences, MA, USA).

### Cell viability assay

Lapatinib-sensitive and lapatinib-resistant cells were mixed in different proportions (percentage of resistant cells per probe was: 0; 5; 10; 25; 50; 75 or 100 %). Combinations of SKBR-3 cells and their lapatinib-resistant counterparts (total concentration: 2 × 10^3^ per well in 100 µl of medium) were seeded into 96-well plate and incubated at 37 °C and 5 % CO_2_ for 24 h. After that time, the medium was removed and fresh medium containing either lapatinib (100 nM), one of ITCs (2.5 µM sulforaphene, 5 µM sulforaphane or 5 µM erucin) or combinations of compounds were added. After 48 h cells were incubated with 26 µl of MTT (4 mg/ml) for additional 3 h. Then, the medium was removed and formazan crystals were dissolved in 100 μl of DMSO. Absorbance (570 and 660 nm) was measured in Victor3 Multilabel Counter (PerkinElmer, USA). Each dose of ITC and lapatinib was tested in triplicate and the experiment was repeated twice.

### Western blotting

Drug-sensitive and drug-resistant SKBR-3 cells were mixed in different proportions and seeded in 4 ml of medium in 6-cm plates. After 24 h, the medium was removed and replaced with a new one, containing lapatinib (100 nM), erucin (5 µM) or combination of compounds. After 48 h, cells were collected and cell lysates, SDS-PAGE electrophoresis and immunoblotting were performed as described previously [18]. To estimate the intracellular level of VEGF protein, cells were seeded in 8 ml of medium in 10-cm plates and allowed to attach for 24-h and treated with erucin, the drug or their combination for 96 h. The intensity of the immunoreactive bands was determined by densitometric scanning to quantify changes in protein levels. Each protein was detected at least twice, from independently prepared lysates.

### Migration assay

1 × 10^6^ cells were seeded in 4 ml of medium in 6 cm plates and a wound was made after 3 days, when cells achieved 100 % confluence as described by Burk [31]. Cells were washed twice with PBS and a fresh medium containing 5 µM erucin and/or 100 nM lapatinib was added. After incubation for 7 days (the medium was exchanged every 2 days) cells were photographed using a light microscope. Experiment was performed three times. Migration was quantified by measuring the distance that cells migrated from the wound edge, at three positions of each photo taken from five randomly selected fields.

### Invasion assay

The effect of erucin–lapatinib treatment, as well as each of this agent alone, on in vitro invasion was determined using BD BioCoat™ BD MatrigelTM Invasion Chamber according to the manufacturer’s instructions. Briefly, after rehydratation of the chambers with serum-free medium for 2 h at 37 °C, 6 × 10^4^ cells were seeded in serum-free medium supplemented with the tested agents or DMSO in each matrigel-coated insert containing membrane with an 8 µm pore size. The lower compartment of the chamber contained 0.75 ml of medium with chemoattractant (10 % FBS). After 48 h, matrigel and non invading cells were removed; invading cells were fixed with 100 % methanol, stained with crystal violet and counted under light microscope. Experiment was performed in triplicate.

### Statistical analysis

Data were analyzed using GraphPad Prism software. Differences between groups in migration and invasion tests were analyzed using Student’s *t* test. Difference was considered significant at *P* <0.05. Differences between groups in viability tests were analyzed using two-way ANOVA followed by Bonferroni’s multiple comparison test.

## Results

### The effect of lapatinib, isothiocyanates and their combinations on viability of drug-sensitive and drug-resistant SKBR-3 cells

**Fig. 1:**
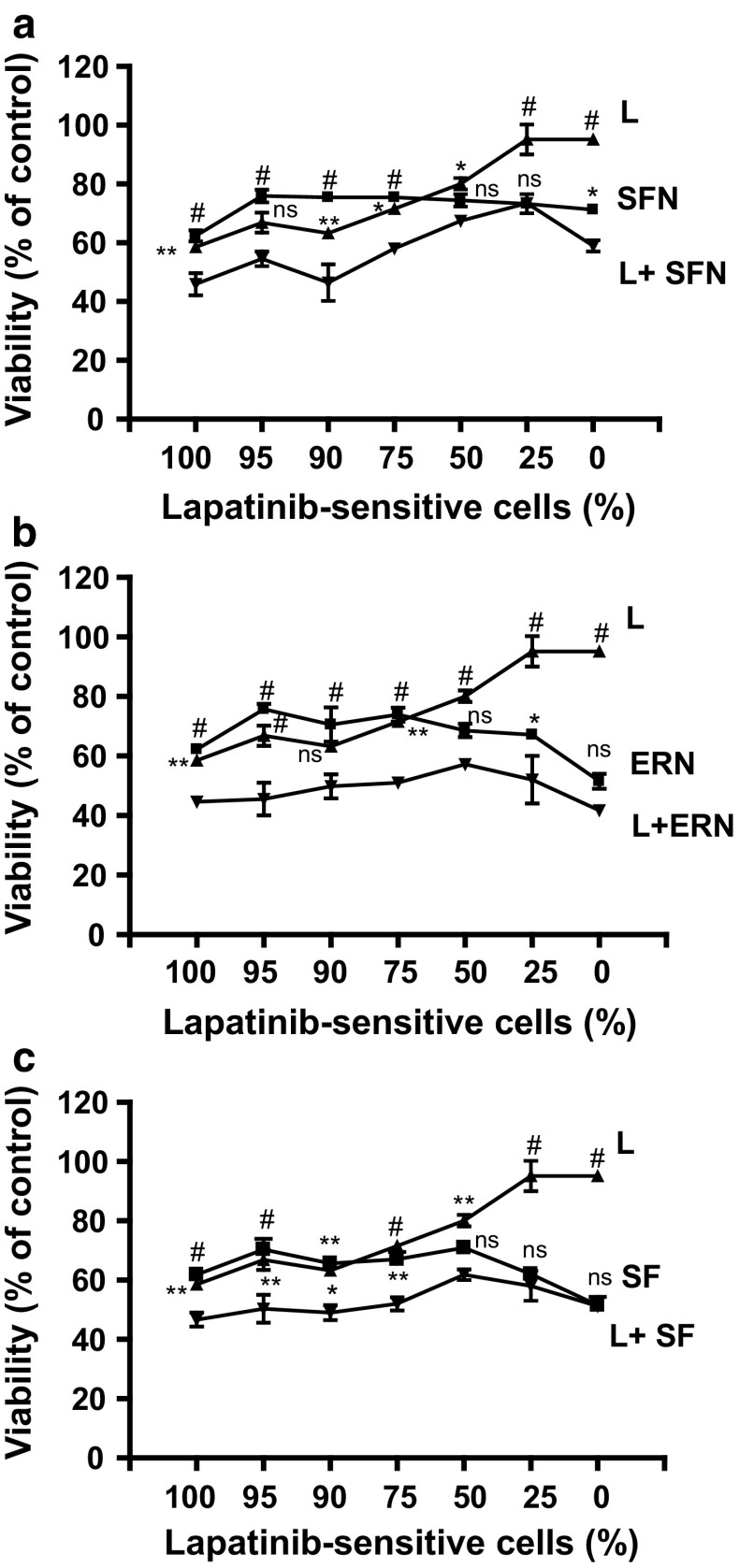
Isothiocyanates sensitize SKBR-3 cells to lapatinib. Lapatinib-sensitive cells were mixed with lapatinib-resistant cells in various ratios (percentage of lapatinib-sensitive cells in a given sample is shown below the graph) and treated for 48 h with 5 μM sulforaphane (SFN—**a**), 5 μM erucin (ERN—**b**), 2.5 μM sulforaphene (SF—**c**), 100 nM lapatinib (L) or combinations. Cell viability was estimated by MTT assay. Each point is mean (±SE) of two experiments done in triplicate (*error bars* are not shown when they are smaller than the symbols). Significant differences between single agent treatment and combination treatment for a given population are indicated as follows: *P < 0.05; **P < 0.01; ^#^ *P* < 0.001; *ns* not significant (two-way ANOVA followed by Bonferroni’s multiple comparison test)

To investigate the influence of combined treatment on viability of cell populations consisting of different proportions of lapatinib-sensitive and lapatinib-resistant cells we used the MTT assay. We chose low doses of isothiocyanates (close to their IC_50_) and the drug (below IC_50_ for the sensitive cell line) in combinations, which have been shown previously to act in a synergistic way [18]. Our model showed that in populations with high percentage of sensitive cells lapatinib was almost as effective as a low dose of an isothiocyanate, however, combined therapy was the most effective (Fig. 1). When percentage of resistant cells increased, lapatinib alone was less effective but low viability level was sustained due to isothiocyanates activity, either alone or in combination with lapatinib. Combination of lapatinib and erucin was the most efficient in cancer cell viability inhibition, even in the drug-resistant population (Fig. 1b).

### Apoptosis induction and cell signaling analysis upon lapatinib-erucin treatment

**Fig. 2:**
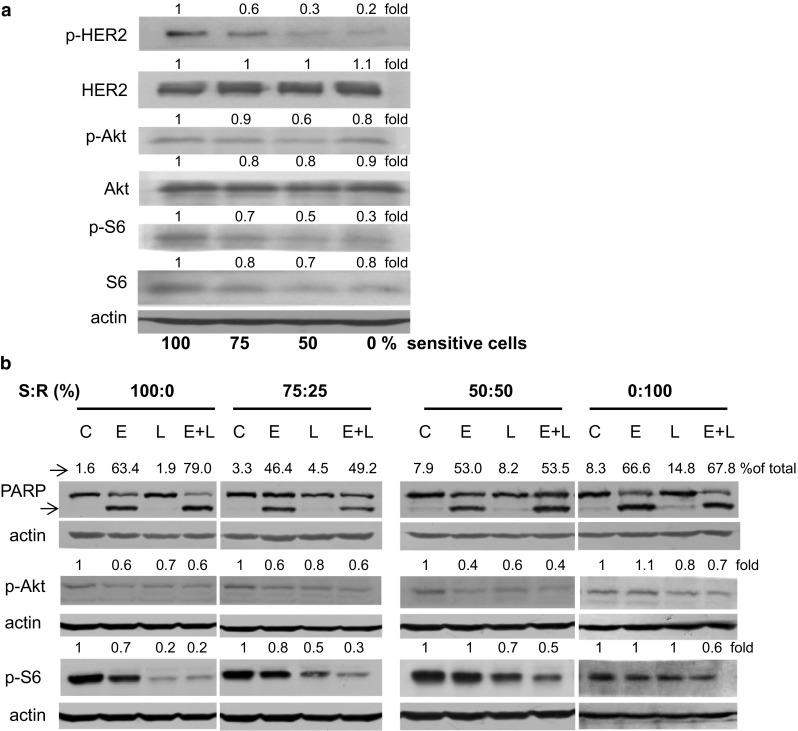
**a** Comparison of the levels and phosphorylation of HER2, Akt and S6 in populations of lapatinib-sensitive and/or lapatinib-resistant cells. **b** Impact of erucin, lapatinib or the combination on PARP cleavage as well as phosphorylation of Akt and ribosomal S6 in cell populations composed of lapatinib-sensitive and lapatinib-resistant SKBR-3 cells. Lapatinib-sensitive and lapatinib-resistant cells mixed in ratio shown above blots (S:R) were treated with 5 μM erucin (E), 100 nM lapatinib (L) or combination of both compounds for 48 h. *Blots* were stripped and reprobed with anti-β-actin antibody to verify equal protein loading. Densitometric analysis data after correction for loading control and relative to control are on *top* of the respective bands. In case of PARP, the percentage of cleaved band in the whole amount of PARP is shown. Similar results were observed in replicate experiments

To get insight into the mechanism involved in lapatinib resistance acquisition we compared status of crucial members of HER2 signaling pathway in non treated drug-sensitive or drug-resistant SKBR-3 cells. As shown in Fig. 2a, in lapatinib-resistant cells the basal level of p-HER2 was much lower than in sensitive cells while the total level of the receptor was similar. The main HER2 downstream effector, the Akt kinase, was slightly decreased in populations enriched in lapatinib-resistant cells. On the other hand, the phosphorylation of ribosomal protein S6, substrate of mTOR-S6K1 was significantly lower in the drug resistant population which was accompanied by a drop in a total level of this protein (Fig. 2a).

To determinate the mechanism of anti-proliferative activity of the combination treatment we used the most effective pair, lapatinib and erucin, and looked at their effect on the apoptosis induction (PARP cleavage) and signal transduction pathway downstream of HER2 (p-Akt and p-S6). In the case of samples exclusively consisting of sensitive cells, we observed the highest percentage of cleaved PARP when cells were exposed to combined therapy, as compared to those treated either with erucin or lapatinib alone which is consistent with previously published results [18]. When the percentage of resistant cells was increased, PARP cleavage was induced mostly due to erucin activity (Fig. 2b).

The drop in the level of phosphorylated Akt kinase was comparable between erucin and erucin with lapatinib-treated cells, although in case of fully drug-resistant cells combination of compounds more efficiently decreased p-Akt than single compounds (Fig. 2b). We also observed that in the case of sensitive cells, the level of the phosphorylated ribosomal protein S6 (p-S6) was reduced by both erucin and the drug, and although in other samples, where proportion of sensitive to resistant cells was 3:1, 1:1 or 0:1, lapatinib or erucin activities were lower, combined treatment still caused the most effective decrease of p-S6 level comparing with effects of single compounds (Fig. 2b).

### Inhibition of motility and invasion of the drug-sensitive and drug-resistant SKBR-3 cells by the combination of erucin and lapatinib

**Fig. 3:**
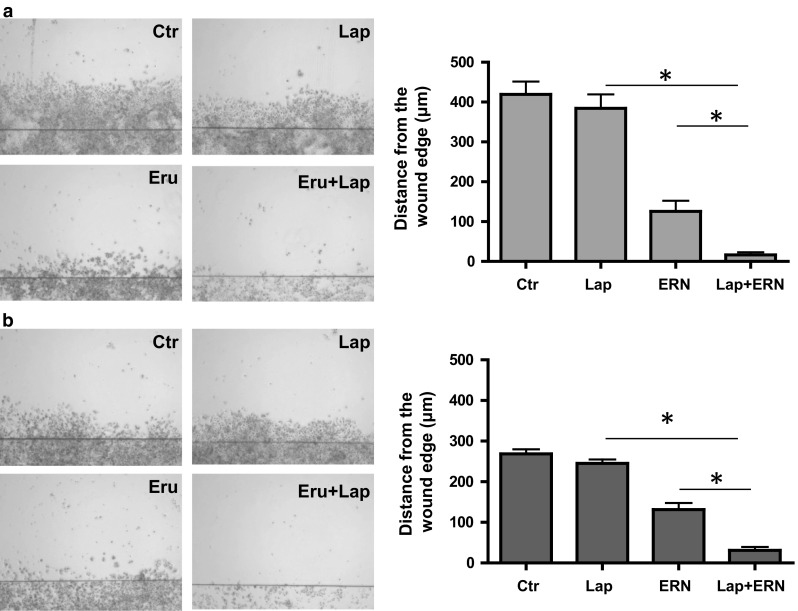
Combination of lapatinib with erucin significantly inhibits cell migration as compared to activity of each agent alone. Confluent monolayers of lapatinib-sensitive (**a)** or lapatinib-resistant (**b**) SKBR-3 cells were wounded with a razor blade. After 7 days, migration through the wound edge was examined under the microscope. Magnification ×4. Data represent the mean ± SE of three independent experiments, in each five randomly chosen fields were examined; *P < 0.05

As Akt-mTOR pathway regulates cancer cell migration and invasion, we investigated whether the combined treatment impacts cell motility using wound-healing assay. Effect of lapatinib, at used by us concentration, on cell migration was weak, irrespective of percentage of the drug resistant cells in the population. However, we noticed that combinations of lapatinib with sulforaphane, erucin or sulforaphene significantly inhibited cell migration as compared to activity of each agent alone (data not shown). We observed the most effective cell motility inhibition for the combination of lapatinib with erucin, both in lapatinib-sensitive (Fig. 3a) and lapatinib-resistant (Fig. 3b) SKBR-3 cells.

**Fig. 4:**
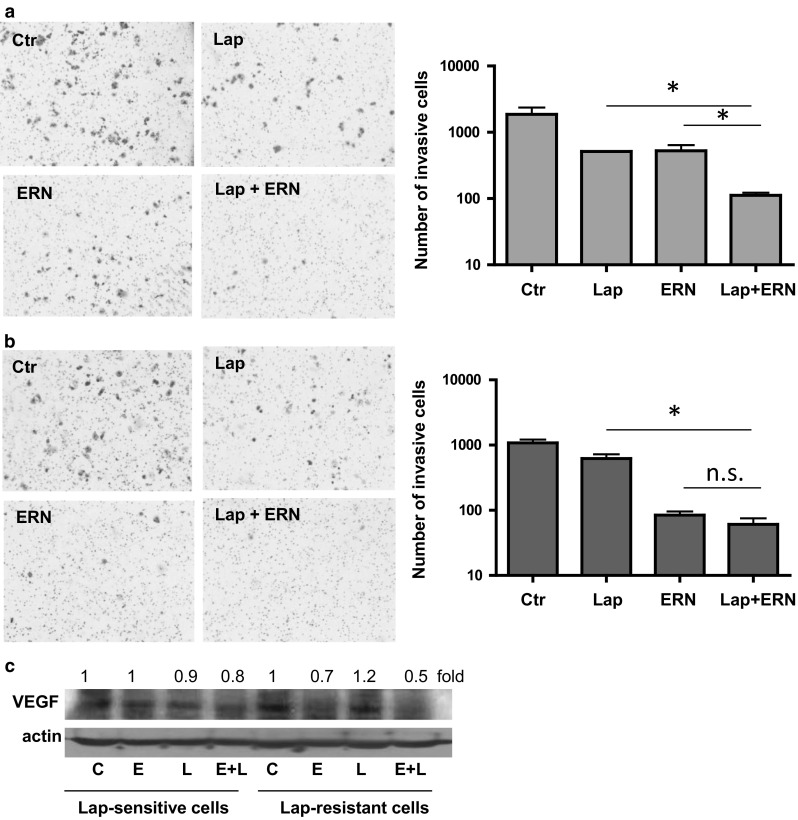
Impact of mono- and combined treatment on invasion and VEGF level in SKBR-3 cells. Combination of lapatinib with erucin inhibits cell invasion of both, lapatinib-sensitive (**a**) and lapatinib-resistant (**b**) SKBR-3 cells. Boyden chamber assay was performed after 48-h treatment, and invaded cells were counted. Data represent the mean ± SE of three parallel experiments; *P < 0.05, *ns* non significant. **c** VEGF level is efficiently reduced by combination of lapatinib with erucin. Cells were treated for 96 h with 5 μM erucin (E), 100 nM lapatinib (L) or combination of both compounds. *Blots* were stripped and reprobed with anti-β-actin antibody to ensure equal protein loading. Densitometric analysis data after correction for loading control and relative to respective controls are on* top* of bands

To evaluate the invasive potential of the drug-sensitive and the drug-resistant SKBR-3 breast cancer cells in response to erucin, lapatinib and combined treatment, we used a transwell matrigel invasion assay. We determined, that combined treatment most efficiently decreased invasiveness of both tested cell lines, as compared to the effect of erucin and the drug alone, however, statistical significance was achieved only in the case of the lapatinib-sensitive cells (Fig. 4). In case of lapatinib-resistant cells, the invasiveness inhibition was caused mostly by erucin (Fig. 4b).

As VEGF is one of the most important regulators of angiogenesis, a complex process that may facilitate metastasis, we verified its level in cells treated with erucin, lapatinib or both agents. Our findings indicate that erucin-lapatinib combination more efficiently decreased the VEGF level in drug-resistant than drug-sensitive cells (Fig. 4c).

## Discussion

Lapatinib is one of the most efficient drugs against HER2-positive breast cancer cells, and one of the most frequently used in therapy at the advanced, metastatic stage. By blocking intracellular ATP-binding site of the receptors, (HER2 and EGFR) lapatinib inhibits activation of both Ras/Raf/MEK/ERK and PI3K/Akt/mTOR/S6K signal transduction pathways, and consequently, induces cell cycle arrest in the G1 phase and apoptosis [32]. Lapatinib inhibits the receptor for a longer time in comparison to other anti-EGFR drugs, e.g., erlotinib (Tarceva) and gefitinib (Iressa), due to its long dissociation half life that is more than 300 min. This drug shows activity in case of breast cancer cell lines that are resistant to trastuzumab. Lapatinib decreases activation of S6K and IGF-1, two signaling pathways that are considered as mediators in trastuzumab resistance [4].

Despite the great success of lapatinib, its use in therapy often becomes ineffective and after incipient tumor regression, recurrence of the disease is observed. This progression is due to primary or acquired resistance [12]. During long-term lapatinib therapy, on the one hand drug-sensitive cells are eliminated; on the other hand probability of drug-resistant cell selection increases [13]. Several mechanisms of acquisition of resistance to lapatinib have been proposed, e.g., mutations within genes of HER2 or EGFR receptors, increased signal transduction from estrogen receptor, overexpression of progesterone receptor, hyperactivation of PIK3Ca or mutations in genes of the PI3K pathway [11, 12]. Lapatinib also derepresses FOXO3a, which stimulates transcription of estrogen receptor (ER) and leads to co-dependence on HER2 and ER signaling [33].

One of the potential ways to overcome drug resistance is a therapy that uses combination of several anti-HER2 drugs, nonetheless lapatinib-based therapy has adverse events (rash, diarrhea, cold symptoms, vomiting, anorexia, nausea, fatigue, headache, gastrointestinal symptoms and hepatotoxicity) [4, 34]. This problem is even more serious in case of treatment using two or more drugs. For example, preclinical studies have shown that combination of lapatinib with trastuzumab more efficiently downregulates survivin and induces apoptosis, than each of these agents alone. However, trastuzumab may impair cardiac function by causing myocardium damage and despite the fact that the mechanism of this cardiac dysfunction is not known, it has been suggested that HER2 may play a protective role for cardiomyocytes [35]. Thus, side effects are the main limitation of such combined therapy.

In this work, we developed cell-based model of lapatinib resistance using HER2- overproducing SKBR-3 cells that were cultured for a few months in the presence of increasing drug concentrations. IC50 for the parental cell line was 120 nM, while their lapatinib-resistant derivatives were able to grow in the presence of more than 600 nM drug. Resistance was not associated with the loss of HER2 expression or sensitivity to lapatinib because phosphorylation of its downstream effectors, such as Akt, was still inhibited in resistant cells. It indicates that resistance occurred by an alternative pathway, similarly as has been previously observed in the case of lapatinib-resistant BT474 cells [36].

We further worked on mixed cell populations consisting of the drug-sensitive and the drug-resistant cancer cells in various proportions which might mimic heterogeneity of breast tumor in vivo in context of the resistance acquisition process. We tested efficiency of anticancer activity of lapatinib, one of isothiocyanates (sulforaphane, erucin or sulforaphene) and their combinations applied in relatively low concentrations which have been previously shown to work in a synergistic way and inhibit downstream elements of the HER-2 pathway in lapatinib-sensitive cells [18]. Our findings support hypothesis that isothiocyanates sensitize lapatinib-resistant cells to the drug. Even when percentage of the resistant cells increased, and drug sensitivity decreased, combination of lapatinib with any of the isothiocyanates tested caused a decline in cell viability and migration potential, as compared to activity of each agent alone.

Interestingly, relatively poorly known erucin in combination with lapatinib was more potent that other combinations. Thus, molecular mechanism was investigated in more details for this combination. The apoptosis marker, cleaved PARP, was increased in cells treated with lapatinib-erucin combination although when the percentage of resistant cells increased, erucin was mostly responsible for apoptosis induction. The analysis also revealed that the combination of lapatinib with erucin, similarly as in the drug-sensitive cells, downregulated PI3K-Akt-mTOR-S6K pathway, which was evidenced by a decrease in p-AKT and p-S6. This is an important observation as it has been reported that PI3K-Akt-mTOR-S6K1 signaling plays prominent roles in apoptosis suppression, drug resistance and metastasis. Alterations in this pathway in breast cancers are often caused by mutations or aberrant expression of numerous genes, including: *HER2*, *BRCA1*, *BRCA2*, *EGFR1*, *ER*α, *PTEN*, *PI3K*, *TP53*, *RB* [37, 38]. S6K1 is one of the regulators of cancer cell invasion, migration and metastasis. It was demonstrated in a model of metastasis of triple-negative breast cancer that S6K1 promotes invasiveness [39]. Moreover, team led by Dihua Yu has shown that ErbB2 (HER-2) increases VEGF protein production by activating S6K in cell lines, xenografts and in human cancers [40]. This observation underscored suggestion that S6K activity may serve as a target for antiangiogenic and antimetastatic therapies [40]. In our model, combination of lapatinib with erucin inhibits S6K more efficiently than treatment with single agents, as revealed by a drop in phosphorylation of its main substrate, the ribosomal S6 protein, which might serve as a marker of successful treatment. Reduced activity of S6K might contribute to the observed decrease in intracellular VEGF level, as well as inhibition of motility and invasiveness of SKBR-3 cells. It is worth mentioning that the combined treatment effectively decreased VEGF and metastatic potential in drug-resistant cells, although effect on invasive potential was mostly exerted by erucin.

In conclusion, the obtained results suggest that application of isothiocyanates, especially erucin, as adjuvant agents during lapatinib therapy is reasonable. This novel solution may not only inhibit resistance acquisition and metastasis processes, but may also be less deleterious for patients as it uses a low dose of each agent. However, to fully understand the potential of this method, further in vitro and in vivo experiments are required.
